# Phenotypic and immune functional profiling of patients with suspected Mendelian Susceptibility to Mycobacterial Disease in South Africa

**DOI:** 10.1186/s12865-021-00452-6

**Published:** 2021-09-13

**Authors:** Ansia van Coller, Brigitte Glanzmann, Helena Cornelissen, Marlo Möller, Craig Kinnear, Monika Esser, Richard Glashoff

**Affiliations:** 1 Immunology Unit, Division of Medical Microbiology, National Health Laboratory Service and Faculty of Medicine and Health Sciences, Stellenbosch University, Tygerberg Hospital, Cape Town, South Africa; 2 DSI-NRF Centre of Excellence for Biomedical Tuberculosis Research, South African Medical Research Council Centre for Tuberculosis Research, Division of Molecular Biology and Human Genetics, Faculty of Medicine and Health Sciences, Stellenbosch University, Cape Town, South Africa; 3 South African Medical Research Council Genomics Centre, Cape Town, South Africa; 4 Division of Haematopathology, National Health Laboratory Services and Faculty of Medicine and Health Sciences, Stellenbosch University, Tygerberg Hospital, Cape Town, South Africa; 5 Department of Paediatrics and Child Health, Faculty of Medicine and Health Sciences, Stellenbosch University, Cape Town, South Africa

**Keywords:** Mendelian Susceptibility to Mycobacterial Disease, Tuberculosis, Immune profiling, IFN-γ-IL-12 cytokine pathways

## Abstract

**Background:**

Mendelian Susceptibility to Mycobacterial Disease (MSMD) is a primary immunodeficiency (PID) characterised by a predisposition to infection by weakly-pathogenic mycobacteria. In countries with a high prevalence of tuberculosis (TB), individuals with MSMD are also prone to infections by *Mycobacterium tuberculosis*. Several MSMD-associated genes have been described, all resulting in a disruption of IL-12 and IFN-γ cytokine axis, which is essential for control of mycobacterial infections. An accurate molecular diagnosis, confirmed by phenotypic and functional immune investigations, is essential to ensure that the patient receives optimal treatment and prophylaxis for infections. The aim of this study was to implement a set of functional assays to assess the integrity of the IL-12-IFN-γ cytokine pathways in patients presenting with severe, persistent, unusual and/or recurrent TB, mycobacterial infections or other clinical MSMD-defining infections such as *Salmonella.*

**Methods:**

Blood was collected for subsequent PBMC isolation from 16 participants with MSMD-like clinical phenotypes. A set of flow cytometry (phenotype and signalling integrity) and ELISA-based (cytokine production) functional assays were implemented to assess the integrity of the IL-12-IFN-γ pathway.

**Results:**

The combination of the three assays for the assessment of the integrity of the IL-12-IFN-γ pathway was successful in identifying immune deficits in the IL-12-IFN-γ pathway in all of the participants included in this study.

**Conclusions:**

The data presented here emphasise the importance of investigating PID and TB susceptibility in TB endemic regions such as South Africa as MSMD and other previously described PIDs relating to TB susceptibility may present differently in such regions. It is therefore important to have access to in vitro functional investigations to better understand the immune function of these individuals. Although functional assays alone are unlikely to always provide a clear diagnosis, they do give an overview of the integrity of the IL-12-IFN-γ pathway. It would be beneficial to apply these assays routinely to patients with suspected PID relating to mycobacterial susceptibility. A molecular diagnosis with confirmed functional impairment paves the way for targeted treatment and improved disease management options for these patients.

**Supplementary Information:**

The online version contains supplementary material available at 10.1186/s12865-021-00452-6.

## Introduction

South Africa is among the countries with the highest burden of tuberculosis (TB) [1] and primary immunodeficiencies (PIDs) that relate to susceptibility to mycobacterial infection, such as Mendelian Susceptibility to Mycobacterial Disease (MSMD), are of particular relevance. MSMD is defined by selective susceptibility to poorly pathogenic mycobacteria such as the Bacille Calmette-Guérin (BCG) vaccine as well as environmental mycobacteria in patients who are otherwise considered healthy [2, 3]. In regions with high prevalence of TB it has been reported that these patients can also present with very severe, persistent, unusual or recurrent (SPUR) *Mycobacterium tuberculosis* (Mtb) infections [2–9].

**Fig. 1:**
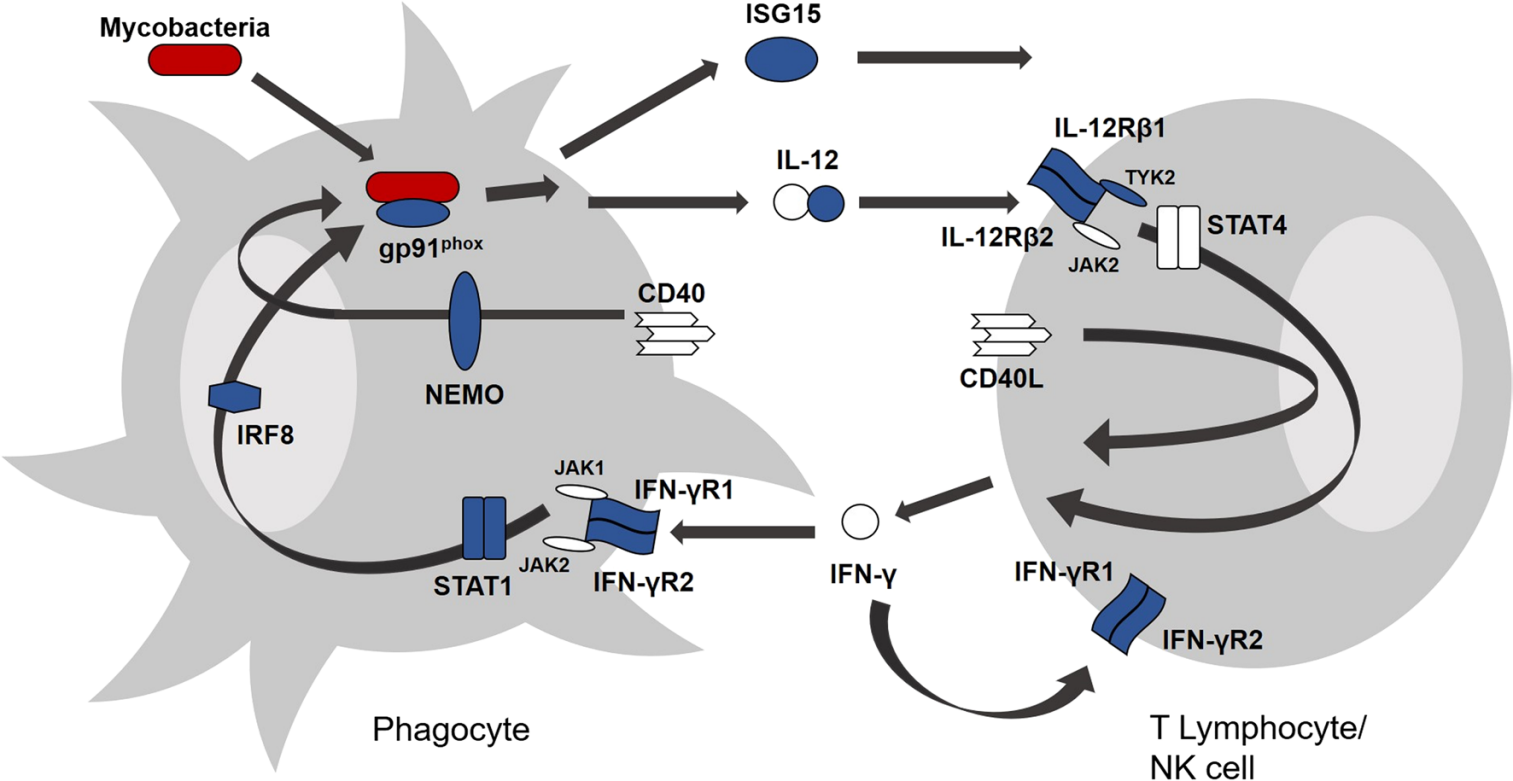
The IL-12-IFN-γ cytokine pathways. The IFN-γ-IL-12 pathway is involved in the host response to infection with certain intracellular viral and bacterial pathogens such as Mycobacteria and *Salmonella.* Upon recognition of the pathogen (indicated in red), phagocytes (such as macrophages and dendritic cells (DCs)) secrete IL-12 which bind to their receptors on T lymphocytes and NK cells, inducing the production of IFN-γ. IL-12 production is enhanced by a CD40-triggered, NEMO/NFκB-dependent pathway. The proteins encoded by genes that have been associated with MSMD are indicated in blue. Mutations in *IFNGR1*, *IFNGR2*, *CYBB*, *STAT1, JAK1* and *IRF8* impair the action of IFN-γ while mutations in *IL12B*, *IL12RB1, IL12RB2*, *ISG15*, *IKBKG* (NEMO) and *IRF8* impairs the function of IFN-γ. (Adapted from Bustamante et al. [2]). *RORC*, *SPPL2A*, *L23R* and their protein products are not indicated in this figure but have also been implicated in MSMD and IFN-γ-mediated immunity)

Fifteen MSMD-associated genes have been described, and mutations in these MSMD-associated genes are associated with a disruption in IFN-γ immunity, which is essential for the control of mycobacterial infections [2, 3, 6, 9–16]. The heterogeneity of these genes result in at least 30 distinct disorders, which vary in their mode of inheritance and clinical presentation [12–14]. All MSMD-associated defects impair IFN-γ immunity by either hindering IFN-γ production or by causing abnormal responses to IFN-γ [13, 17–20]. The IFN-γ-IL-12 pathway and the MSMD-associated mutations within this pathway are illustrated in Fig. 1.

Treatment of MSMD needs to be targeted to the specific disease-causing genetic defect and clinical presentation [21]. Therefore, patients with suspected MSMD, including those presenting with SPUR Mtb infections, should undergo molecular testing to diagnose their immune deficit as this can impact on clinical outcomes and responses to therapy [22, 23]. However, it is estimated that about half of MSMD patients lack a defined genetic aetiology [24]. Established infections in MSMD patients are generally treated with the appropriate antimicrobials, and prophylactic antimicrobials are administered, even in periods of disease remission [21], but the delay in diagnosis can significantly hinder the proper treatment for the affected patients.

In countries with a high TB burden, it is essential to identify individuals with MSMD since they could acquire early and potentially devastating Mtb infections if they are not diagnosed timeously [25]. It is possible that MSMD and other PIDs that are associated with TB susceptibility are more common in South Africa than previously believed due to these PIDs being overshadowed by the massive HIV-TB co-epidemics [26].

Currently, the most effective and reliable way of diagnosing rare PIDs is by means of next generation sequencing (NGS); however, the resulting data can be very time consuming to process and interpret [6, 27–29]. Even where a candidate disease-causing variant has been identified by NGS, functional validation tests still need to be done in order to confirm the manifestation of the genetic defect in the patients’ immune cells [28]. Rapid and functional-driven screening assays can aid in the diagnosis of PIDs associated with TB susceptibility such as MSMD in countries like South Africa with a high endemicity of TB.

The aim of this study was to implement a set of functional assays to assess the integrity of the IL-12-IFN-γ cytokine pathways in patients with suspected MSMD or TB-related PID. The overall goal was to determine whether the combination of these functional assays, which have each been used previously in isolation to help diagnose defects in the IL-12-IFN-γ cytokine pathways, could be applied to aid in the diagnosis of MSMD or TB-related PID in South Africa. The functional results from well-described MSMD mutations have been previously summarised by Esteve-Sole’ et al. [30].

## Methodology

### Study participants

Patients with suspected MSMD, i.e. presenting with severe, persistent, unusual and/or recurrent (SPUR) TB, mycobacterial infections or other clinical MSMD-defining infections such as *Salmonella* were recruited to the study over a period of five years (September 2013–July 2018). Patients were recruited through the paediatric immunology clinic at Tygerberg academic hospital in the Western Cape of South Africa. A severe infection was defined as an infection that was uncontrolled or complicated—e.g. TB empyema, TB pericarditis, TB meningitis, TB spine, multiple osteoarticular sites of TB, other disseminated forms of TB including miliary TB. Persistent infections were defined as infections that required longer duration of therapy (> 6 or > 9 months depending on severity) or had a lack of response to appropriate treatment regimen for > 2 months. Unusual was defined as infections at an unusual or unexpected site, e.g. dissemination of TB to ears, spine/brain, liver, osteoarticular TB, etc., or an unusual organism, e.g. *M. avium* or BCG which only cause disease in patients with compromised immune systems; and recurrence was defined as two or more occurrences of the same infection (at least one year apart) despite completion of therapy and subsequent elimination of previous infections.

Only individuals under the age of 15 years and older individuals who had their first relevant clinical presentation before the age of 15 years were included in this study. All the patients underwent standard clinical examinations (including analysis of a medical and family history, vaccination and infection history, physical examination with a focus on assessment of any dysmorphic features, evaluation of lymph nodes and rashes, etc.) and routine laboratory testing (including HIV tests, conventional TB testing including the Qiagen QuantiFERON TB IFN-γ release assay (QFT), full blood counts and differential subsets enumeration, enumeration of immunoglobulins, Neutrophil burst assay, etc.) to exclude other illnesses or causes of immunodeficiency.

Blood was collected by venepuncture into ethylenediaminetetraacetic acid (EDTA) tubes and peripheral blood mononuclear cells (PBMCs) were isolated using the density-gradient centrifugation method [31]. PBMCs were cryopreserved in liquid nitrogen. Blood plasma was also stored at – 80 °C.

An additional MSMD patient from our setting presenting with both pulmonary TB and disseminated *M. avium* infection, with a well described heterozygous, partial dominant mutation in *IFNGR1* (c.818del4), was included as a control for the functional assays [32]. PBMCs were also collected from 10 self-reported healthy volunteers (ages: 22–45 years) to serve as controls for this study.

### Cytokine-induced cytokine production assay

The assay used in this study to assess the levels of IL-12-induced IFN-γ production and vice versa was derived from the IFN-γ and IL-12 production assays with BCG/Phytohaemagglutinin (PHA) co-stimulation developed by Feinberg et al. [33] and Dorman and Holland [30, 34].

All recombinant human cytokines used in this study were procured from BD Biosciences (CA, USA). PHA, PenStrep and Foetal Bovine Serum (FBS) was procured from Sigma-Aldrich® (MO, USA).

Cryopreserved PBMCs were thawed, washed with Roswell Park Memorial Institute medium (RPMI-1640 with 1% PenStrep and 10% FBS) and counted on a Bio-Rad TC20™ cell counter (Bio-Rad Laboratories, CA, USA) to determine cell concentration and viability. Four wells in a 24-well cell culture plate were prepared for each patient: (1) no activation (NIL), (2) PHA activation (5 µg/mL), (3) PHA activation and IL-12 stimulation (20 ng/mL), and (4) PHA activation and IFN-γ stimulation (50 ng/mL). Between 150,000 and 500,000 cells were added per well (with > 90% viability), depending on availability. The exact number of cells added into each well was recorded in order to adjust cytokine concentrations to represent cytokine production per 10^5^ PBMCs. The plate was then incubated for 48 h at 37 °C with 5% CO_2_. After incubation, the contents of each well were centrifuged at 2,500 RCF for 10 min and the supernatants were harvested.

IFN-γ and IL-12p70 concentration was determined through Enzyme-linked Immunosorbent assay (ELISA) using Quantikine® ELISA for human IFN-γ and Quantikine® HS ELISA for human IL-12p70 (Qiagen, Hilden, Germany), according to manufacturer’s instructions. Concentrations of each cytokine were expressed as pg/mL cytokine produced by the equivalent of 10^5^ PBMCs:

$$[\text{cytokine produced per }{10}^{5}\text{ cells}\left(\frac{{\text{pg}}}{{\text{mL}}}\right)=\frac{{\text{total cytokine concentration }\left(\frac{{\text{pg}}}{{\text{mL}}}\right)\text{measured by ELISA}}}{{\text{number of cells per well}}} \times {10}^{5}]$$

Net IL-12 production induced by IFN-γ and net IFN-γ production induced by IL-12 was determined for each individual using the calculated concentrations of cytokines produced per 10^5^ PBMCs in each of the respective wells.

IFN-γ concentration present in blood plasma at the time of PBMC isolation was also determined for each patient using the same Quantikine® ELISA kit for human IFN-γ.

### Assessment of cytokine receptor expression and signalling through Flow Cytometry

All monoclonal antibodies, recombinant human cytokines, and Phosflow™ reagents used in this study were procured from BD Biosciences (CA, USA). Flow cytometric data acquisition was performed on a BD LSR II (BD Biosciences, CA, USA) at the BD-CAF Flow Cytometry Centre at Stellenbosch University, Tygerberg Medical Campus.

#### Cytokine receptor detection through standard surface flow cytometry

Assessment of IFN-γR1 (CD119) and IL-12Rβ1 (CD212) expression has previously been used to confirm several cases of MSMD and the method used in this study was adapted from these previous studies [30, 35–38]. For cytokine receptor detection, cryopreserved PBMCs were thawed and washed with RPMI (with 1% PenStrep and 10% FBS), and then split into two separate polypropylene tubes, with each tube containing at least 5 × 10^5^ cells (with > 90% viability). One of the tubes were left unstimulated, for IFN-γR1 detection, and 5 µg/ mL PHA was added to the other, for upregulation of IL-12Rβ1 [39]. Both tubes were placed in a 5% CO_2_ incubator at 37 °C for 24 h. After the incubation period, the PBMCs were washed twice, first with RPMI (with 1% PenStrep and 10% FBS) and then with staining buffer (Phosphate buffered saline [PBS; Sigma-Aldrich®, MO, USA] with 2% FBS).

Surface expression of IFN-γR1 (CD119) and IL-12Rβ1 (CD212) on lymphocyte subsets, NK cells and monocytes were then evaluated by standard surface flow cytometry, whereby cells were stained with the following fluorochrome-conjugated monoclonal antibodies at the specified dilutions: 1:20 CD3-FITC (UCHT1), 1:160 CD4-APC (SK3), 1:40 CD8-PE-Cy5 (HIT8a), 1:40 CD14-PE-Cy7 (M5E2), 1:20 CD56-BV510 (R19-760), 1:160 CD19-BB700 (HIB19), 1:20 CD119-PE (GIR-208), 1:20 CD212-BV421 (2-4E6)] and 1:2000 FVS575V (a fixable, amine reactive viability dye).

#### Assessment of cytokine signalling through phospho-specific intracellular flow cytometry

IFN-γ and IL-12 signalling was assessed by measuring the intracellular phosphorylation of the respective cytokine receptor-associated signal transducer molecules STAT1 and STAT4. This was achieved by an intracellular flow cytometric-based phosphorylation assay—Phosflow™—where, after PBMC stimulation with either recombinant human IFN-γ or IL-12 cytokines, levels of phosphorylated STAT1 and STAT4 (pSTAT1 and pSTAT4) were measured intracellularly by detecting the bound phospho-specific fluorochrome-conjugated antibodies. Similar methods for the detection of pSTAT1 and pSTAT4 have previously been used to assess IFN-γ and IL-12 signalling in several MSMD cases [30, 36, 40].

Cryopreserved PBMCs were thawed and washed with RPMI (with 1% PenStrep and 10% FBS), and split into three separate polypropylene tubes (labelled unstimulated, IFN-γ stimulated, and IL-12 stimulated), with each containing at least 5 × 10^5^ cells (with > 90% viability). PHA (5 µg/mL) was added to the IL-12 stimulated tube—to upregulate IL-12Rβ1 expression for optimal pSTAT4 detection following IL-12 stimulation. The samples were then placed in a 5% CO_2_ incubator at 37 °C for ± 18 h. After the incubation period, the PBMCs were washed with RPMI (with 1% PenStrep and 10% FBS). The cells were subsequently stained with the fixable viability marker, FVS575V.

Cells were then stimulated with 500 µL of either RPMI (unstimulated), 100 ng/mL human recombinant IFN-γ (IFN-γ stimulated) or 100 ng/mL human recombinant IL-12 (PHA pre-stimulated tube) for 15 min at 37 °C, 5% CO_2_. Immediately after stimulation, the cells were fixed using BD Cytofix™. Thereafter, BD Perm IV (1X) was used for permeabilisation of cells.

Each tube was then stained with an antibody cocktail containing the following antibodies at the specified dilutions: 1:20 CD3-FITC (UCHT1), 1:160 CD4-APC (SK3), 1:40 CD8-PE-Cy5 (HIT8a), 1:40 CD14-PE-Cy7 (M5E2), 1:20 CD56-BV510 (R19-760), 1:10 CD20-PerCP-Cy5.5 (H1), 1:20 pSTAT1-BV421 (4a), and 1:5 pSTAT4-PE (38-pSTAT4).

### Data analysis

For the receptor panel, the frequency (%) of CD119 and CD212 expression for each of the parent populations were measured as well as the density or median fluorescence intensity (MFI) of expression on each cell subset. For the signaling panel, the fold change in pSTAT detection between the cytokine stimulated and unstimulated samples for each patient was determined for both pSTAT1 and pSTAT4. Fold change in pSTAT detection was calculated as the MFI of cytokine stimulated sample divided by the MFI of unstimulated sample.

The ELISA data and the flow cytometric data [analysed in FlowJo® version 10.6.2 (©Becton Dickinson & Company)] were exported into Microsoft Excel files for statistical analysis. Medians and 95% confidence intervals (CI) were used to describe the non-parametric data. The 95% CI was used as a ‘normal’ range against which patient data could be compared to on an individual basis.

### Genetic testing

Following the functional assays, the patients were referred for whole exome sequencing (WES) through the Primary Immunodeficiencies Genetics Network (PIDDGEN) research group at Stellenbosch University. Sequencing was carried out on the Ion Proton™ (Thermo Fisher, Carlsbad, California, United States) at the Central Analytical Facility (CAF) at Stellenbosch University, Stellenbosch, South Africa. Sequences were aligned to the human reference genome, hg19 (https://www.ncbi.nlm.nih.gov/assembly/GCF_000001405.13/) using TMA (version 5.10) in the ion-analysis workflow on the Torrent Suite (version 5.10). The variant caller (version 5.10) plugin on the Torrent suite was used for base quality score recalibration, indel realignment and variant calling. TAPER™, a custom-designed, in-house method was used for variant prioritization and variants in a homozygous recessive state or compound heterozygous state were ranked as potential candidate variants [27].

Various in-silico prediction tools were used to estimate the likelihood of pathogenicity of the identified variants. Sorting intolerant from tolerant (SIFT) [41], PolyPhen [42], MutationTaster [43] and Combined Annotation Dependant Depletion (CADD) Scores [44] were all used. Thereafter The American College of Medical Genetics and Genomics (ACMG) criteria [45] and the newer Sherloc criteria, which is a refined version of the ACMG criteria, [46] were used to make a final call on the pathogenicity of each variant.

Candidate variants identified through WES were confirmed by Sanger sequencing using the BigDye® Terminator v3.1 Cycle Sequencing Kit (Perkin-Elmer, Applied Biosystems Inc., CA, USA.), followed by electrophoresis on an ABI 3130XL Genetic Analyzer (Perkin-Elmer, Applied Biosystems Inc., CA, USA). All automated DNA sequencing reactions were performed at CAF at Stellenbosch University, Stellenbosch, South Africa.

## Results

### Study participants

Fifteen patients with suspected MSMD were enrolled in this study, as well as one patient with a known, well-described MSMD mutation (*IFNGR1* [c.818del4]). Of the 16 patients included in this study, 10 (63%) patients had multiple TB episodes, and 12 (80%) had disseminated or extrapulmonary TB. Non-tuberculous mycobacterial infections were confirmed in only 1 case, PID01 (*M. avium*). All participants received BCG vaccination as infants, although none presented with suspected BCG dissemination. One patient presented with *Salmonella* infections (PID04). None of the patients had a family history of confirmed PID. One of the patients, PID08, had a sibling with a similar clinical presentation who had died in infancy. Another patient, PID16, had a history of severe TB on the paternal side of the family. Some of the participants had household TB contacts from an early age, although most had no known TB contacts in their home environment at the time of recruitment to this study.

There was no gender bias amongst the participants, 8 (50%) were male and 8 (50%) were female. The onset of clinical disease presentation ranged between 3 weeks of age and 15 years old (average age of 3.5 years), while the average age at enrolment to this study was 8 years old (range 1 year to 23 years old). A summary of the clinical presentation and relevant routine laboratory investigations can be found in Table 1.

**Table 1:** Clinical information for the 16 patients enrolled in the study

Patient	Sex	Clinical Presentation	Age at first presentation	Age at recruitment to study	Family history	IFN-γ release assay (QFT)	Routine Lab results (Immunoglobulins, Complement, Neutrophil burst, Full blood count & differential subsets)
PID01 *IFNGR1* (c.818del4)	F	Concurrent pulmonary TB, TB lymphadenitis and recurrent disseminated *M.avium* infection	6 months	23 years	No relevant PID history	Not performed	Routine Immunological workup normal
PID02	F	Diagnosed with pulmonary TB and TBM (Stage III) at age 9	9 years	10 years	TB exposed (mom). Younger sib also developed TBM	Not performed	Routine Immunological workup not done in full*. All investigations done were normal
PID03	F	Recurrent pulmonary TB (3 episodes), including MDR TB, as well as TB lymphadenitis	5 years	10 years	No relevant PID history. Household TB contact (mom)	Not performed	Slightly elevated IgA and IgM (raised less than 2 SD). All other investigations done were normal
PID04	M	Chronic mucoid diarrhoea since 3 weeks of age. Recurrent *Salmonella* type C infections	3 weeks	3 years	No relevant PID history	Not performed	All routine lab investigations normal. Autoimmune investigations normal
PID05	M	Recurrent pulmonary TB—4 culture confirmed cases, each more than 1 year apart. Previously diagnosed with Goldberg-Shprintzen Syndrome	9 months	14 years	No relevant PID history	Positive	Routine Immunological workup not done in full*. All investigations done were normal
PID06	M	Unusual extrapulmonary TB with spine and liver involvement	11 years	12 years	No relevant PID history. Household TB contacts	Not performed	Anaemia. Routine Immunological workup not done in full*. All investigations done were normal
PID07	M	Recurrent TB lymphadenitis requiring prolonged duration of treatment	3 years	4 years	No relevant PID history	Positive	Routine Immunological workup not done in full*. All investigations done were normal
PID08	F	Miliary TB and TB lymphadenitis	1 year	1 year	Sibling died early in childhood, with similar clinical presentation	Negative	Anaemia. Routine Immunological workup not done in full*. All investigations done were normal
PID09	F	Recurrent TB of the liver (6 + episodes). Shortly after end of treatment, TB reactivates, and patient needs to restart treatment. The most recent TB episode was MDR TB	1 year	7 years	No relevant PID history. MDR TB household contact	Not performed	Routine Immunological workup normal
PID10	M	Recurrent TB. First episode was miliary TB and 6 months after end of treatment presented with TBM. Second TBM episode (Stage II) 8 months after completion of TB treatment for previous episode	3 months	3 years	No relevant PID history	Not performed	Routine Immunological workup normal
PID11	F	This patient had a new-born that was diagnosed with TB. Upon investigation it was revealed that she (the mother) has had 6 suspected, although 4 confirmed, and treated episodes of pulmonary TB	15 years	20 years	No relevant PID history	Negative	Routine Immunological workup normal
PID12	M	Recurrent TB abscess of anterior chest wall	2 years	10 years	No relevant PID history. Household TB contacts	Positive	Routine Immunological workup normal
PID13	M	Septic arthritis and miliary TB, which progressed to TBM and eventual MDR TB regime	11 months	1 year	No relevant PID history	Not performed	Anaemia. Routine Immunological workup normal
PID14	F	Unusual TBM diagnosis in 9-year-old	9 years	10 years	No relevant PID history	Not performed	Routine Immunological workup normal
PID15	F	Recurrent TB, with hip involvement, and MDR TB	1 year	6 years	No relevant PID history	Positive	Routine Immunological workup normal
PID16	M	Recurrent TB (4 episodes), including MDR TB	6 months	5 years	History of severe& recurrent TB on paternal side	Positive	Routine Immunological workup normal

*Neutrophil burst assay not performed for all patients due to difficulties related to sample collection from the individuals

### IFN-γ-IL-12 pathway functionality

The results from the functional assays were highly variable between the patients, with each patient having a unique functional readout. All patients showed a defect in at least 2 aspects of the assays. A short summary of the functional data for the major cell subsets for each patient can be found in Table 2. Composite data sets for IFN-γR1 and IL-12Rβ1 percentage positive and receptor density (MFI) expression, fold changes in pSTAT1 and pSTAT4 detection, as well as the IL-12-induced IFN-γ and IFN-γ-induced IL-12 production results for each patient as well as the control 95% Confidence intervals can be found in Additional file 1: Tables S1–S5.

**Table 2:**
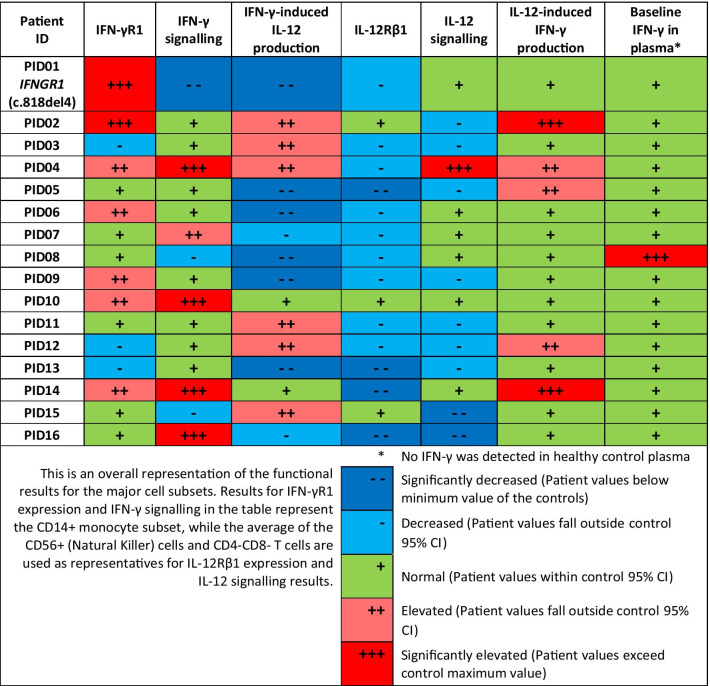
Summary of functional results for all 16 patients

PID01, the previously diagnosed MSMD patient with a mutation in *IFNGR1* (c.818del4), showed very high levels of IFN-γR1 expression but contrastingly had very low levels of IFN-γ signalling (pSTAT1 fold change) through the receptor as well as reduced IL-12 production upon IFN-γ stimulation. PID01 also had decreased levels of IL-12Rβ1 expression, although IL-12 signalling and IL-12-induced IFN-γ production was comparable to the controls.

IL-12Rβ1 expression on CD56+ (NK) cells and CD4-CD8- T cells were decreased for 13 (81%) of the 16 suspected MSMD patients, while 10 (63%) patients had abnormal IFN-γR1 expression. Significantly elevated IFN-γR1 expression was seen in PID01 and PID02, while three other patients had slightly reduced IFN-γR1 expression compared to the controls.

**Fig. 2:**
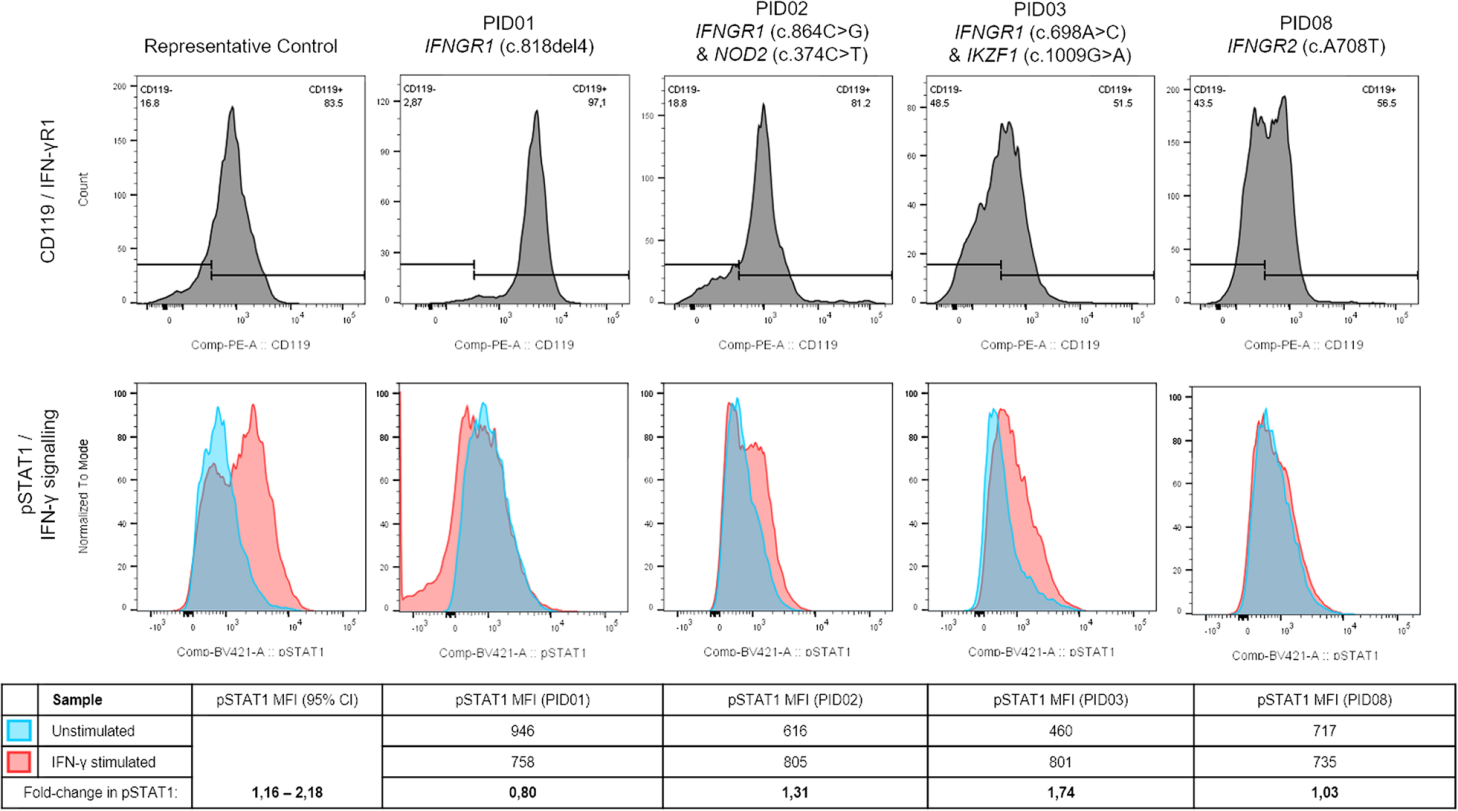
IFN-γR1 detection and IFN-γ signalling in CD14 + monocytes in control and suspected MSMD patients. PID01, who has a known mutation in *IFNGR1* (c.818del4), had elevated levels of IFN-γR1 expression, but decreased levels of IFN-γ signalling (fold-change in pSTAT1 detection following stimulation with IFN-γ) compared to the controls. Other patients for whom variants have been identified in IFN-γR are also shown in the figure—these suspected MSMD patients showed varying degrees of IFN-γR1 expression and IFN-γ signalling

**Fig. 3:**
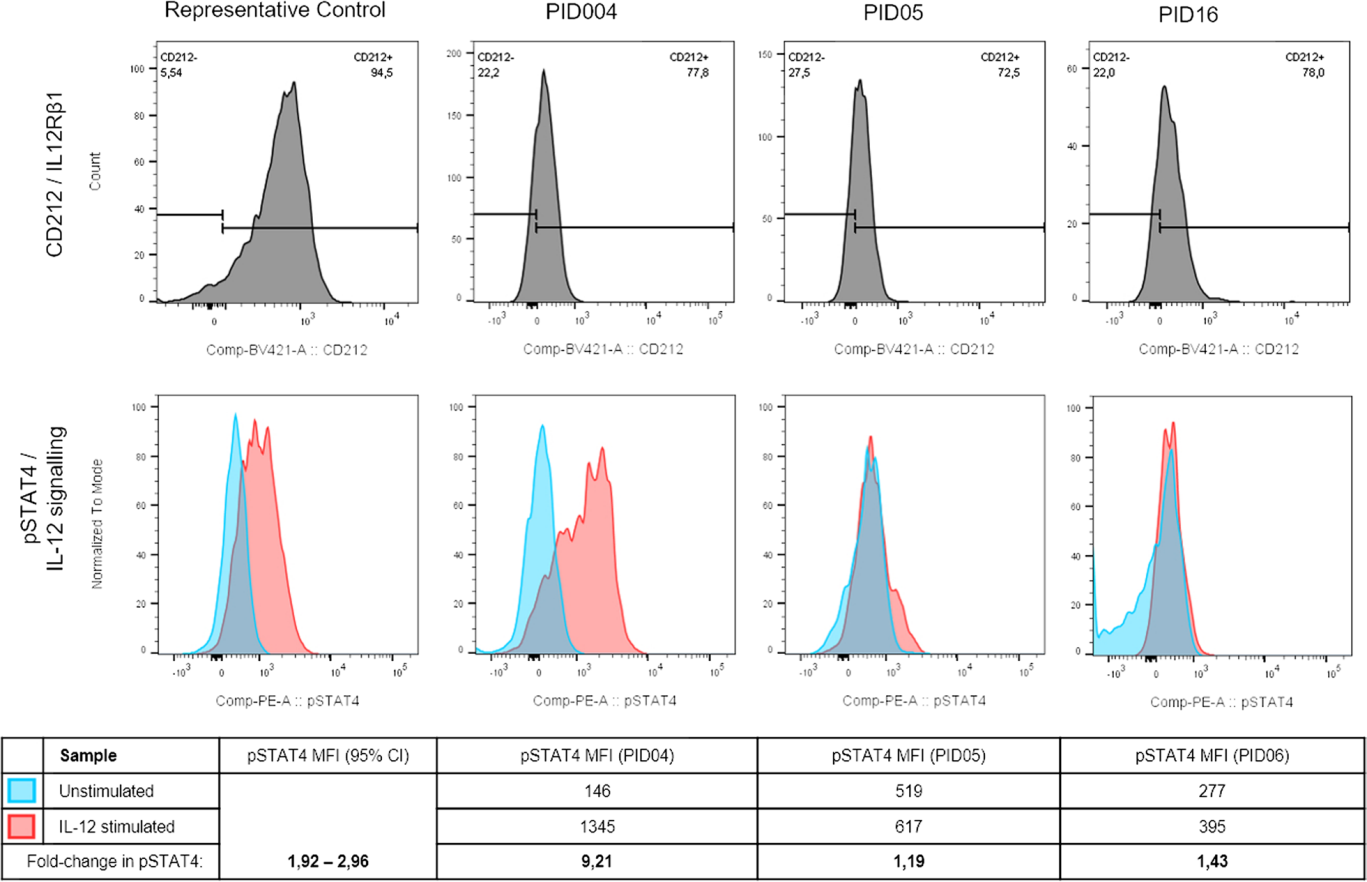
IL-12Rβ1 expression and IL-12 signalling in CD56 + NK cells in control and suspected MSMD patients. PID04, PID05, and PID16 all had low levels of IL-12Rβ1 expression compared to controls. Additionally, PID05 and PID06 had decreased levels of IL-12 signalling (fold-change in pSTAT4 following IL-12 stimulation) compared to controls, but PID04 contrastingly had elevated IL-12 signalling

**Fig. 4:**
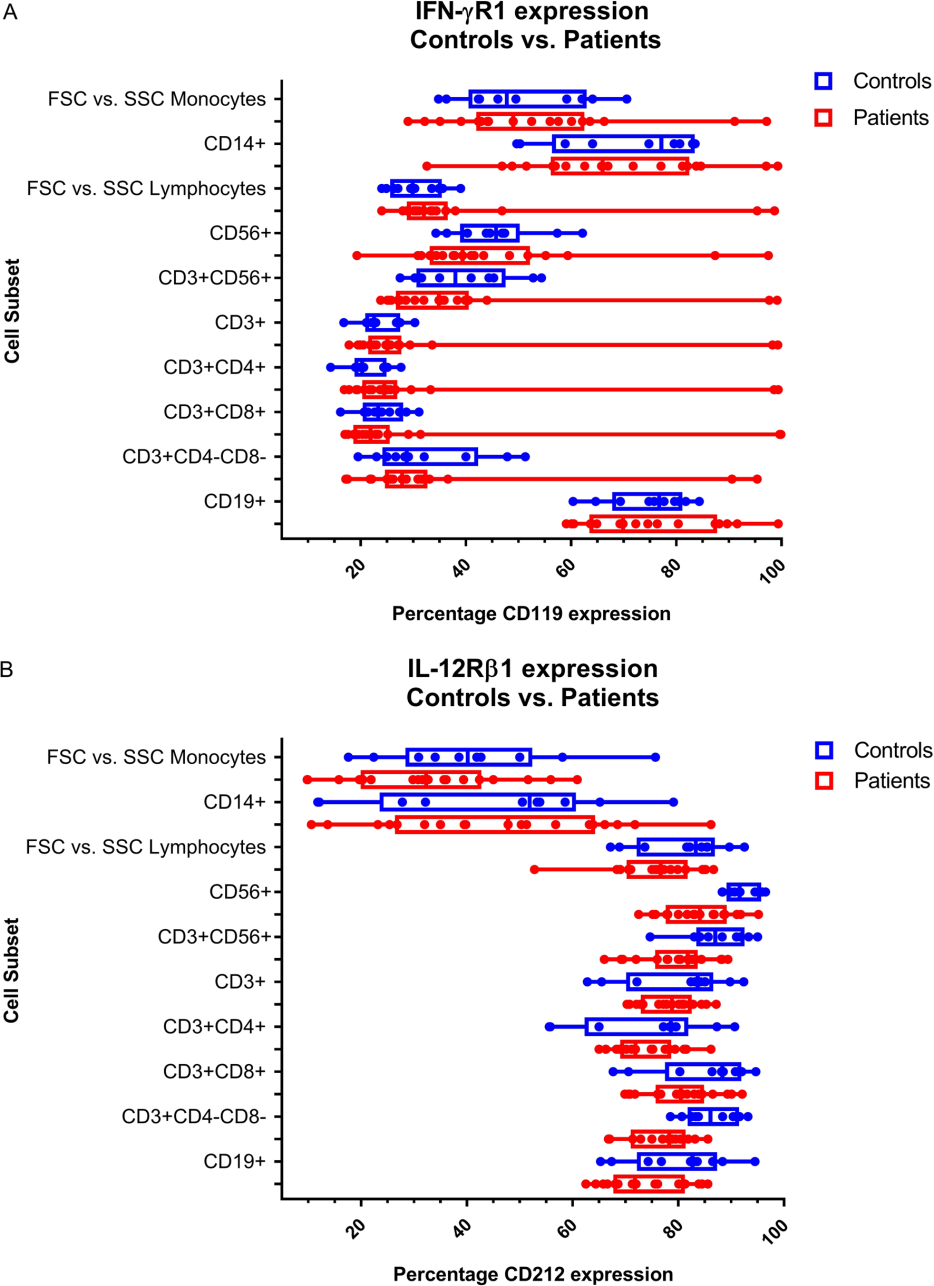
Comparison of distribution of cytokine receptor expression levels for controls (n = 10) and suspected MSMD patients. **A** IFN-γR/CD119 percentage expression on unstimulated PBMCs for controls and patients on various cell subsets; **B** IL-12Rβ1/CD212, percentage expression on PHA pre-stimulated PBMCs for controls and patients on various cell subsets. The box-and-whiskers plot shows minimum and maximum values at the ends. The 50% confidence interval (25th to 75th percentile) are the borders of the box, with the middle line representing the median. Each dot represents a patient or control value. There were no significant differences in receptor expression levels between the controls and the patient group, however, the patient results should rather be considered on a case-by-case manner due to the diversity in clinical presentation of the patients

**Fig. 5:**
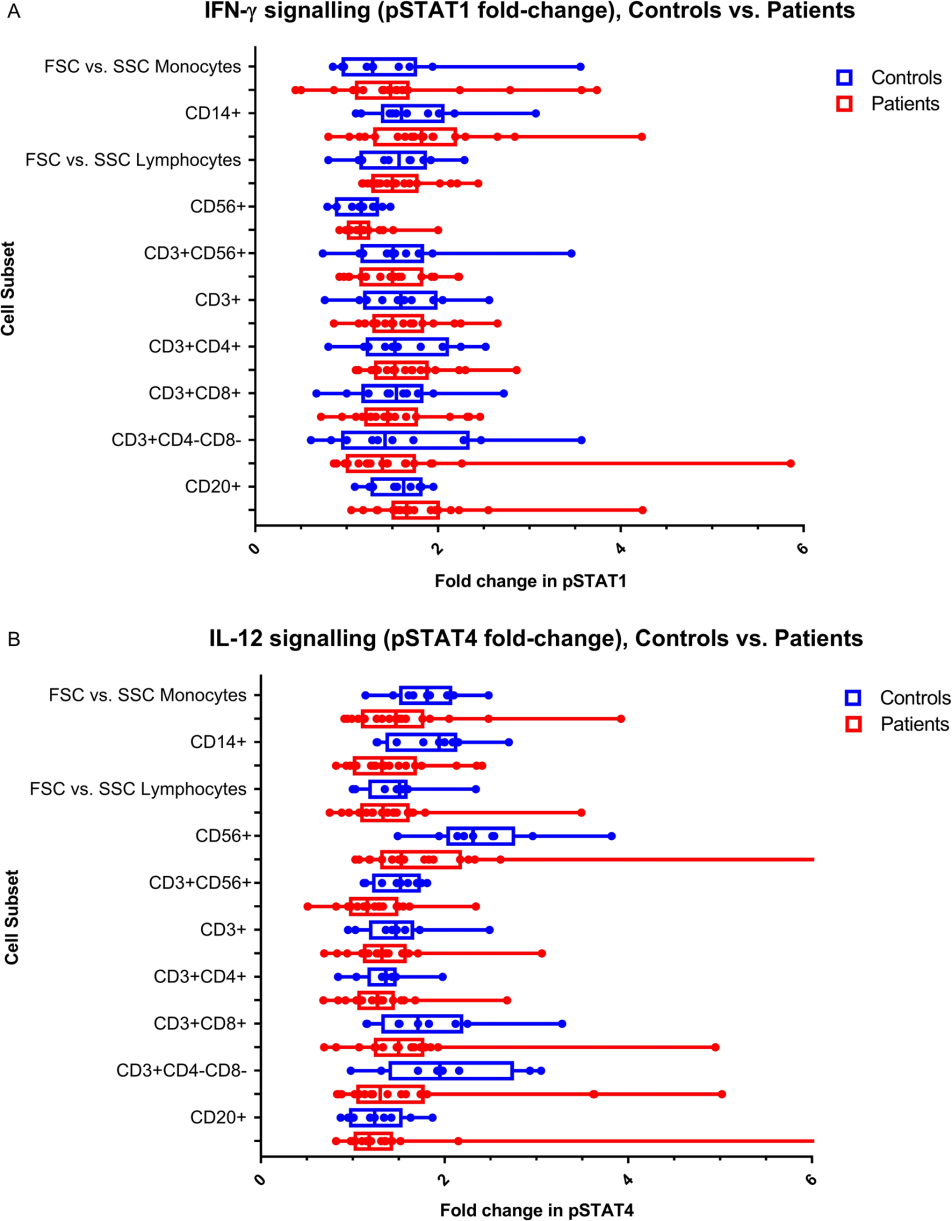
Comparison of distribution of fold changes in pSTATs for controls (n = 10) and patients. **A** Fold change in pSTAT1, i.e. IFN-γ signalling, for controls and patients in various cell subsets. pSTAT1 was measured for both unstimulated and IFN-γ (100 ng/mL) stimulated PBMCs; **B** Fold change in pSTAT4, i.e. IL-12 signalling, for controls and patients in various cell subsets. pSTAT4 was measured on PHA pre-stimulated PBMCs that were then either left unstimulated or stimulated with IL-12 (100 ng/mL). There were no significant differences in cytokine signalling between the controls and the patient group, however, the patient results should rather be considered on a case-by-case manner due to the diversity in clinical presentation of the patients

**Fig. 6:**
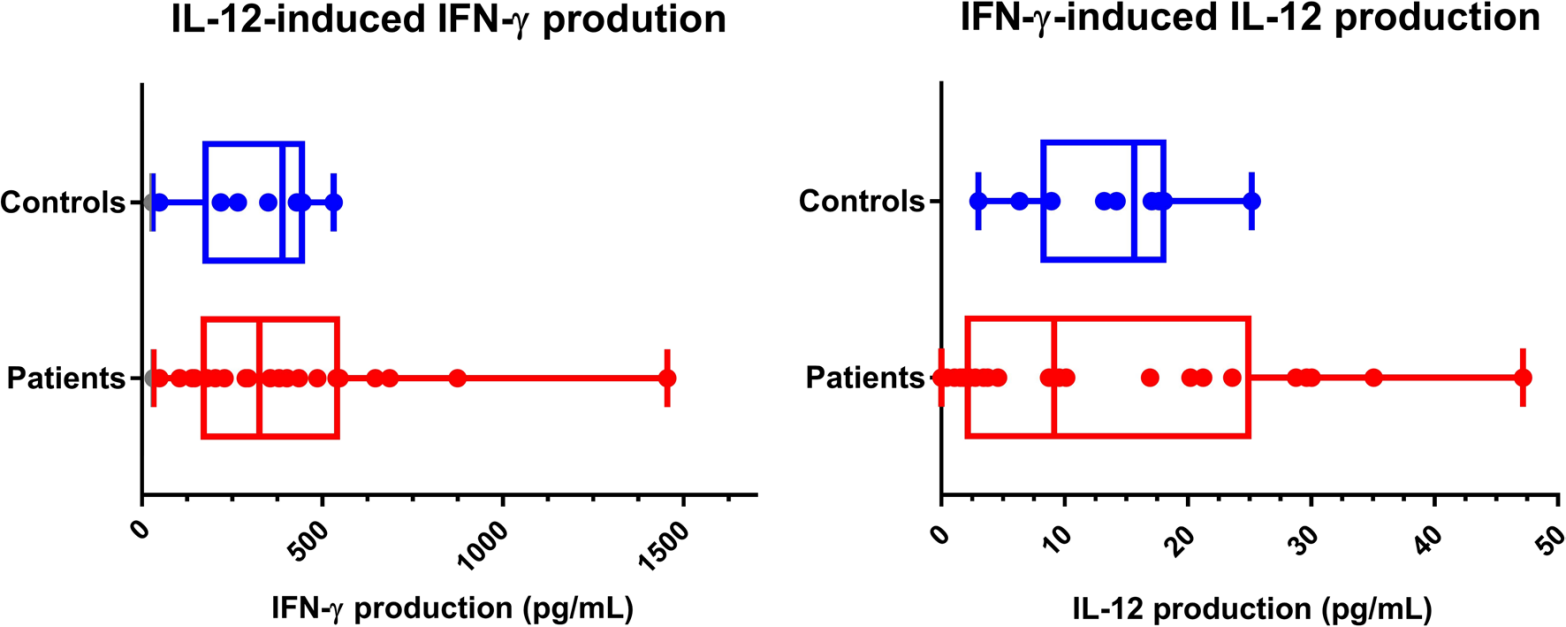
Comparison of distribution of cytokine-induced cytokine production for controls and patients. Cytokine production was measured per 100,000 PBMCs. **A** IL-12-induced IFN-γ production. The patient group has a larger range, with several patients falling outside the IQR of the control group; **B** IFN-γ-induced IL-12 production. Several patients fall outside the control IQR, with an apparent clustering of high and low IL-12 producers. There were no significant differences in cytokine production levels between the controls and the patient group, however, the patient results should rather be considered on a case-by-case manner due to the diversity in clinical presentation of the patients

Figure 2 shows a visual representation of IFN-γR1 expression and IFN-γ signalling in CD14+ monocytes for a representative control as well as for PID01 [with the known *IFNGR1* (c.818del4) mutation] and two other suspected MSMD patients with potential IFN-γR-related deficiencies. Figure 3 shows the levels of IL-12Rβ1 expression and related IL-12 signalling in a representative control and three of the suspected MSMD patients with unusual results. Comparison of cytokine receptor expression levels, cytokine signalling, and induced cytokine production for controls (n = 10) and suspected MSMD patients can be seen in Figs. 4, 5, and 6 respectively.

IFN-γ and IL-12 signalling did not always correlate to the respective receptor expression, for instance the three patients with decreased IFN-γR1 expression had normal levels of IFN-γ signalling and five of the patients with decreased IL-12Rβ1 expression had normal IL-12 signalling, while another patient with decreased IL-12Rβ1 expression (PID04) paradoxically had very high levels of IL-12 signalling.

The cytokine production assays revealed that all of the patients had defective IFN-γ and/or IL-12 production, with little to no IL-12 production observed in 7 patients (excluding PID01), overproduction of IL-12 was observed in 6 patients, while overproduction of IFN-γ was observed in 5 patients. Lack of IFN-γ production was not seen in any of the patients in this study. Only one patient, PID08, had IFN-γ detectable in blood plasma, which was undetectable in the controls and other patients at the time of sample collection.

### Genetics

Variants in known MSMD-associated genes were found in 6 of the 12 sequenced participants. This includes variants in *IFNGR1*, identified for 2 patients (c.698A > C and c.864C > G), *IFNGR2* (c.708A > T) in 1 patient, *IL12B* in 2 patients (c.320A > G and c.863G > C), and *IL12RB2* (c2071A > G) in 1 patient. Other genetic variants, which are currently not known or described as associated with MSMD but could still potentially explain the clinical phenotype, were detected in the other participants. These include *IKZF1* (c.1009G > A), *NOD2* (c.374C > T), *IRAK1* (c.1939C > T), *IKBKB* (c.2257G > A), and *NFKB2* (c.2042C > T). The preliminary genetics data including the transcript ID, Global minor allele frequency and the predicted clinical significance of each identified variant is presented in Table 3. None of the reported genetic variants have been previously associated with MSMD and further investigations are required to determine whether these variants are related to the clinical phenotype observed for each patient.

**Table 3:** Preliminary genetics findings for the suspected MSMD patients

Patient	WES performed?	WES Result- Gene(s) & Variant(s)	rsID	Transcript ID	Zygosity	Global minor allele frequency	Clinical significance (ClinVar)
PID01	N/A	*IFNGR1* (c.818del4)	rs587776856	NM_000416.3	Heterozygous	Unknown	Pathogenic
PID02	Yes	*IFNGR1* (c.864C > G p.Ile288Met)	rs121913184	NM_000416.3	Heterozygous	0.00003594	Not provided
		*NOD2* (c.374C > T p.Pro125Leu)	rs149390911	NM_001370466.1	Heterozygous	0.0002979	Likely benign
PID03	Yes	*IKZF1* (c.1009G > A p.Gly337Ser)	rs148169768	NM_006060.6	Homozygous	0.000717	Not reported in ClinVar
		*IFNGR1* (c.698A > C p.Gly233Ala)	rs767099047	NM_000416.3	Heterozygous	0.000003978	Not reported in ClinVar
PID04	Yes	No result	N/A	N/A	N/A	N/A	N/A
PID05	Yes	No result	N/A	N/A	N/A	N/A	N/A
PID06	Yes	*IL12B* (c.320A > G p.Lys107Arg)	None	NM_002187.3	Heterozygous	Unknown	Not reported in ClinVar
		*NFKB2* (c.2042C > T p.Pro681Leu)	rs534798782	NM_001322934.2	Heterozygous	0.00003211	Not reported in ClinVar
PID07	Yes	*IL12B* (c.863G > C p.Arg288Thr)	None	NM_002187.3	Homozygous	0.00111	Not reported in ClinVar
PID08	Yes	*IFNGR2* (c.708A > T p.Glu236Asp)	rs121913212	NM_005534.4	Homozygous	0.0007108	Variant of unknown significance
PID09	Yes	*IKBKB* (c.2257G > A p.Ala753Thr)	rs200977622	NM_001556.3	Homozygous	0.00001193	Variant of unknown significance
		*NFKB2* (c.2042C > T p.Pro681Leu)	rs534798782	NM_001322934.2	Heterozygous	0.00003211	Not reported in ClinVar
PID10	Yes	*IRAK1* (c.1939C > T p.Leu647Pro)	rs113047476	NM_001569.4	Hemizygous	0.00001974	Not reported in ClinVar
PID11	Yes	*IL12RB2* (c.2071A > G p. Arg691TGly & c. 1259G > A p.Gly420Glu)	rs748575002 & rs765425600	NM_001374259.2	Both Heterozygous	0.000043282 & 0.000003979	Neither reported in ClinVar
PID12	Yes	No result	N/A	N/A	N/A	N/A	N/A
PID13	Yes	No result	N/A	N/A	N/A	N/A	N/A
PID14	No	No result	N/A	N/A	N/A	N/A	N/A
PID15	No	N/A	N/A	N/A	N/A	N/A	N/A
PID16	No	N/A	N/A	N/A	N/A	N/A	N/A

## Discussion

The overall goal of this study was to determine whether the combination of functional assays, which have previously been used in isolation to describe MSMD related defects, could be applied to aid in the diagnosis of suspected MSMD in South Africa where these patients generally have a different clinical presentation (SPUR TB) than what was originally defined as MSMD (BCG and non-tuberculous Mycobacteria).

The most common clinical presentation of patients in the study was severe (disseminated) and recurrent Mtb infections. This is not unexpected, due to the high prevalence of TB in South Africa, however this does differ from what has been described in developed countries where MSMD is usually associated with non-pathogenic mycobacteria [1, 2]. Of the 16 patients in the study, only one presented with non-tuberculous mycobacterial infections, namely PID01 who had concurrent pulmonary TB and disseminated *M. avium* infection [32]. Only one patient, PID04, presented with recurrent *Salmonella* infection, which is a common MSMD presentation associated with mutations in *IL12RB1* and *IL12B* [47], however, no plausible disease-causing variants in either of these genes were identified in this patient through WES.

All the participants were BCG vaccinated, but none presented with BCGosis, which is one of the most commonly reported presentations of MSMD worldwide. To date, BCGosis has not been as commonly reported in South African MSMD cases as compared to those in developed countries [21, 48–50]. It has been previously described that BCG vaccination is not entirely effective at preventing TB infection, although it is still used routinely due to its ability to prevent severe TB and disseminated TB in children [1, 4, 50]. In the current cohort of PID patients, it appears that the BCG vaccination had no protective effect as it did not prevent TB disease or even severe and disseminated forms of TB.

Negative IFN-γ release assay (QFT-plus) results i.e. lack of IFN-γ production in response to TB antigens, in three participants that had confirmed TB episodes (PID03, PID08 and PID11) are a clear indication that there is a defect related to IFN-γ production in response to TB antigenic stimulation. This shows that the routine IFN-γ release assays could be a useful tool to identify potential PID in patients with confirmed TB, although a positive QFT result does not necessarily rule out a PID.

The patients in this study were from a wide range of ages when they had the onset of their symptoms, although the majority had their first manifestations before the age of three years. Unfortunately, the majority of these patients were only investigated for PID/MSMD many years after the onset of their symptoms—highlighting the importance of raising awareness for PIDs, particularly those relating to TB, in South Africa and other TB endemic countries. It is possible that individuals presenting with SPUR TB in South Africa are being dismissed by clinicians, however they could potentially have inherent defects in the IL-12- IFN-γ pathway, which may require specialized treatment. While this study was focused on children with suspected MSMD, it should be noted that several cases presenting in adulthood have been reported in various countries, attributed to the lower penetrance of some MSMD defects [11, 51, 52]. Therefore, MSMD should be suspected at any age if the clinical phenotype is suggestive thereof.

The combination of the three functional assays for the assessment of the integrity of the IL-12-IFN-γ pathway revealed immune deficits in all the participants with suspected MSMD. The results generated by the functional assays were variable, and in several cases the functional data alone cannot fully explain the disease phenotypes; however, even in the absence of genetic data to aid in interpretation, the functional assays provide some clues as to the likely defect.

The three functional assays did not always correlate with each other, for instance, PID05 had lower than normal levels of IL-12Rβ1 expression and IL-12 signalling, however IFN-γ production upon IL-12 stimulation was paradoxically higher than normal. In several cases, the cytokine assay did not correlate well to the cytokine receptor and signalling readouts. This may be an unexpected but still accurate observation of the immune functionality of the immune cells in vitro, however, it could also be due to the non-mycobacterial-specificity of the cytokine assays or the fact that the cytokine production is measured for total PBMCs and not for each respective cell subset as with the flow cytometric assays. The percentages of constituent cell subsets in the PBMCs of each patient may differ and this may have impacted on the final readout of the cytokine assay.

The functional readouts for the *IFNGR1* (c.818del4) patient (PID01) agreed with what has been previously reported. It is known that this variant leads to increased expression of non-functional IFN-γR1 on the surface of immune cells which is unable to transduce a phosphorylation signal following binding of the ligand (IFN-γ) due to lack of intracellular binding sites for signalling molecules [30].

PID09 had a functional readout that was somewhat similar to PID01 in terms of IFN-γR1 expression and IFN-γ-induced IL-12 production although IFN-γ signalling was slightly elevated in PID09, which means that PID09 is unlikely to have the same c.818del4 mutation in *IFNGR1* as PID01. It is more likely that PID09 has a defect downstream of the IFN-γ receptor which may be causing the lack of IL-12 production. Subsequent WES and analysis identified a homozygous variant of unknown significance in *IKBKB* (c.753A > T). A loss of function (LOF) mutation in *IKBKB* has been previously described to be associated with mycobacterial infection (BCG dissemination) and functional work of this LOF mutation showed decreased IL-12 production following IFN-γ stimulation as with PID09 [53]. Further, more specific, functional work will need to be done to confirm the pathogenicity of the identified *IKBKB* variant and its association with MSMD. PID02, like PID01, had high levels of IFN-γR1 expression, however, unlike PID01, the IFN-γ signalling and induced IL-12 production was not reduced while the levels of both IFN-γ and IL-12 production were elevated. It is not clear how the variants identified in PID02 (heterozygous variants in *IFNGR1* [c.864C > G] and *NOD2* [c.374C > T]) relate to the clinical and functional phenotype of this individual.

The majority of the patients had low but detectable levels of IL-12Rβ1 expression although it is not known whether this is due to them all having inborn defects relating to IL-12 receptor. PBMCs were only stimulated for 24 h with PHA prior to detection of IL-12Rβ1, which is much shorter than the 48-h stimulation used by other authors to induce IL-12 receptor expression [33, 34]. Stimulation with PHA for 24 h proved to be sufficient to induce IL-12Rβ1 expression in the control samples that were used to set up the assays, but it is possible that it may not have been sufficient for these patients’ samples; However, in many cases, the patients with decreased IL-12Rβ1 expression also had aberrant IL-12 signalling readouts which supports the theory that these patients’ may have defects relating to IL-12 receptor expression and/or signal transduction. Previously described IL-12Rβ1 mutations in patients with MSMD are usually coupled to complete loss of IL-12Rβ1 expression, which was not seen in any of the patients in this study [30, 39]. Subsequent WES and analysis identified no variants in *IL12RB1* in any of the patients in this study, however, variants of unknown significance were identified in *IL12RB2* in PID11 [*IL12RB2* (p.691A > T and p.420G > E) both heterozygous]. The functional impact of each of these identified variants will need to be investigated further to determine whether they are associated with the functional profiles seen and the clinical presentation of the respective patients.

Dysfunctional responses in the cytokine production assay were observed in several of the patients. Defective IL-12 production following IFN-γ stimulation could indicate a defect in genes associated with production of the IL-12 cytokine (e.g. *IL12B*) or defects in genes relating to the expression and function of the IFN-γ receptor (e.g. *IFNGR1*, *IFNGR2*, or *STAT1*) [2]. PID05, PID06, PID07, PID08, PID13 and PID16 all had low levels of IL-12 production upon PHA and IFN-γ stimulation, although IFN-γ signalling was normal, indicating possible defects relating to *IL12B* or other genes related directly to the expression of IL-12. It could also be possible that these patients have other phagocyte- or lymphocyte-related defects that lead to their PBMCs being less responsive to stimulation. Subsequent WES and analysis by collaborators in the PIDDGEN research group identified likely pathogenic variants for PID06 [*IL12B* (c.320A > G) heterozygous & *NFKB2* (c.2402C > T) heterozygous] and PID07 [*IL12B* (c.863G > C) homozygous]. *IL12B* is a well described etiology of MSMD, and the decreased IL-12 production observed in PID06 and PID07 supports the genetic variants identified in these patients [54]. Further work is needed to define the effect of the *IL12B* and *NFKB2* variants identified in these patients and to determine whether they are associated with the MSMD-like phenotypes observed in these patients.

Overproduction of IFN-γ has been associated with defects relating to the IFN-γ receptor, while lack of IFN-γ production could be due to defects relating to IL-12 receptor functionality or lack of lymphocyte responsiveness [2]. Only one patient, PID08, had IFN-γ present in blood plasma, which has been described in IFN-γ receptor defects [5]. PID08 also had lower than normal levels of IFN-γ signalling and IFN-γ-induced IL-12 production, which is highly indicative of a defect in either IFN-γR1 or IFN-γR2 (which was not measured in this study). Subsequent WES and analysis identified likely pathogenic homozygous variant in *IFNGR2* (c.708A > T) for PID08, which is in agreement with the functional readout observed for this patient.

The defective functional readouts observed for the patients in the study indicate that there are immune deficits in the IL-12-IFN-γ pathways of these patients that could be resulting in their clinical presentation of SPUR TB. These assays could be a useful tool to identify suspected MSMD patients in South Africa, although subsequent genetic investigation would still be required to confirm molecular defect(s). The data from the functional assays can also be used to aid in the interpretation of NGS data (and potentially identify new MSMD-associated genes) as well as advise on clinical management and/or treatment of the affected individuals.

## Conclusion

This study implemented functional immune assays which allowed for the evaluation of the integrity of the IL-12-IFN-γ cytokine pathways in 15 patients with suspected MSMD. This is the first documented study in South Africa with such a diverse cohort of MSMD patients—in terms of clinical presentation—with readouts of immune functionality. While the functional readouts are indicative of dysfunction in the IL-12-IFN-γ pathways of these patients and support the clinical MSMD diagnosis, it should be noted that these data do not necessarily prove the pathogenicity of all candidate variants that were subsequently identified by WES. Further variant-specific research will need to be done for all the candidate disease-causing variants identified in this study to confirm their association with MSMD/PID.

The results obtained in this study emphasise the importance of investigating PID and TB susceptibility in TB endemic regions as MSMD and other previously described PIDs relating to TB susceptibility may present differently in TB endemic areas such as South Africa. It is therefore important to also have access to in vitro functional investigations to better understand the immune function of these individuals.

Although functional assays alone are unlikely to always provide a clear diagnosis, they do give an overview of the integrity of the IL-12-IFN-γ pathway and can support or allude to genetic findings. It would be beneficial to apply these assays routinely to patients with suspected PID relating to mycobacterial susceptibility, particularly in TB endemic regions.

At present, the cost and accessibility of WES and other NGS approaches limit their usefulness in resource limited settings. An alternative may be the use of functional screening assays for MSMD, like the ones implemented in this study, which can identify immune deficits in the IL-12-IFN-γ pathways and inform whether targeted Sanger sequencing can be used to find a molecular diagnosis based on their functional readout, or whether the patient should be referred for further investigation through NGS.

## Supplementary Information

**Additional file 1**. Supplementary data file.

## Data Availability

Majority of the data generated during this study are included in this article and its Additional file 1. All data used and analyzed during the present study will be available from the corresponding author on reasonable request.
