# Supplementary material for: Phenotypic and immune functional profiling of patients with suspected Mendelian Susceptibility to Mycobacterial Disease in South Africa
Source: BMC Immunol. 2021 Sep 13;22:62. doi: 10.1186/s12865-021-00452-6 (PMC8436520; doi:10.1186/s12865-021-00452-6)
Supplement: Supplementary file 1 — Additional file 1. Supplementary data file. [file 12865_2021_452_MOESM1_ESM.docx]

**Table S1: CD119/IFN-γR1 expression levels (%) and receptor densities (MFI) for each of the cell subsets**.

|  | FSC vs. SSC Monocytes | | CD14+ | | FSC vs. SSC Lymphocytes | | CD56+ | | CD3+CD56+ | | CD3+ | | CD4+ | | CD8+ | | CD4-CD8- | | CD19+ | |
| --- | --- | --- | --- | --- | --- | --- | --- | --- | --- | --- | --- | --- | --- | --- | --- | --- | --- | --- | --- | --- |
|  | %CD119+  95% CI:  36-64 | CD119 MFI  95% CI:  305-489 | %CD119+  95% CI:  50-64 | CD119 MFI  95% CI:  620-805 | %CD119+  95% CI:  25-36 | CD119 MFI  95% CI:  64-91 | %CD119+  95% CI:  36-57 | CD119 MFI  95% CI:  51-67 | %CD119+  95% CI:  30-52 | CD119 MFI  95% CI:  93-129 | %CD119+  95% CI:  21-28 | CD119 MFI  95% CI:  68-88 | %CD119+  95% CI:  19-25 | CD119 MFI  95% CI:  61-80 | %CD119+  95% CI:  21-29 | CD119 MFI  95% CI:  97-121 | %CD119+  95% CI:  23-48 | CD119 MFI  95% CI:  47-110 | %CD119+  95% CI:  65-82 | CD119 MFI  95% CI:  98-132 |
| PID01 | 97.20 | 2646 | 97.10 | 5451 | 98.70 | 506 | 97.50 | 328 | 99.20 | 515 | 99.30 | 511 | 99.40 | 482 | 99.90 | 557 | 95.40 | 375 | 99.40 | 707 |
| PID02 | 49.00 | 447 | 81.20 | 1175 | 34.40 | 106 | 34.40 | 45 | 44.00 | 168 | 29.40 | 126 | 29.60 | 108 | 25.20 | 173 | 28.70 | 83 | 80.40 | 164 |
| PID03 | 29.00 | 282 | 51.50 | 588 | 30.40 | 99 | 33.50 | 51 | 32.00 | 122 | 25.10 | 101 | 24.50 | 94 | 21.80 | 137 | 25.10 | 73 | 69.70 | 112 |
| PID04 | 57.60 | 454 | 83.80 | 917 | 33.30 | 97 | 55.20 | 76 | 27.50 | 105 | 24.80 | 98 | 23.70 | 92 | 21.90 | 123 | 30.90 | 71 | 63.90 | 96 |
| PID05 | 42.90 | 523 | 46.90 | 704 | 33.40 | 122 | 30.90 | 67 | 27.10 | 146 | 19.50 | 122 | 17.80 | 103 | 17.60 | 151 | 31.00 | 143 | 64.90 | 134 |
| PID06 | 52.50 | 423 | 71.80 | 820 | 33.70 | 130 | 51.80 | 77 | 35.90 | 150 | 27.10 | 130 | 26.60 | 119 | 25.20 | 157 | 27.90 | 82 | 76.40 | 132 |
| PID07 | 39.10 | 275 | 65.80 | 759 | 28.00 | 100 | 31.50 | 39 | 28.60 | 117 | 22.50 | 105 | 22.20 | 100 | 19.00 | 128 | 26.20 | 67 | 60.50 | 94 |
| PID08 | 60.10 | 423 | 56.50 | 536 | 46.90 | 134 | 59.40 | 100 | 25.80 | 144 | 25.10 | 110 | 24.80 | 99 | 21.00 | 193 | 25.00 | 116 | 89.70 | 211 |
| PID09 | 63.60 | 519 | 84.70 | 827 | 32.00 | 78 | 41.00 | 47 | 35.40 | 100 | 25.70 | 75 | 24.10 | 66 | 22.80 | 106 | 36.60 | 69 | 69.40 | 108 |
| PID10 | 49.00 | 449 | 66.00 | 855 | 29.00 | 79 | 37.90 | 63 | 25.40 | 85 | 20.50 | 77 | 19.20 | 68 | 18.70 | 97 | 32.30 | 80 | 63.70 | 104 |
| PID11 | 56.00 | 382 | 57.00 | 519 | 31.10 | 122 | 43.40 | 56 | 38.40 | 151 | 27.20 | 120 | 26.60 | 109 | 23.40 | 152 | 33.10 | 92 | 87.40 | 168 |
| PID12 | 35.10 | 272 | 32.60 | 394 | 29.20 | 98 | 37.60 | 47 | 30.30 | 119 | 23.00 | 103 | 21.70 | 97 | 20.40 | 129 | 31.60 | 92 | 59.10 | 85 |
| PID13 | 32.20 | 275 | 48.80 | 563 | 30.60 | 100 | 35.60 | 41 | 25.10 | 107 | 24.80 | 100 | 25.70 | 96 | 23.00 | 118 | 21.80 | 70 | 74.60 | 128 |
| PID14 | 66.30 | 655 | 82.10 | 917 | 28.80 | 80 | 33.20 | 45 | 34.90 | 107 | 19.90 | 79 | 19.40 | 76 | 18.90 | 106 | 17.20 | 36 | 72.30 | 110 |
| PID15 | 42.40 | 401 | 59.00 | 745 | 24.00 | 87 | 19.30 | 30 | 39.90 | 129 | 17.80 | 95 | 16.90 | 90 | 17.00 | 125 | 17.50 | 70 | 69.30 | 119 |
| PID16 | 62.20 | 500 | 67.00 | 704 | 30.10 | 73 | 39.40 | 56 | 23.80 | 78 | 21.80 | 72 | 20.70 | 65 | 19.60 | 91 | 25.70 | 66 | 60.10 | 85 |

**Table S2: Fold change in pSTAT1 for each of the cell subsets**.

| **Patient ID** | **FSC vs. SSC Monocytes**  95% CI: 0.95-1.94 | **CD14+**  95% CI: 1.16-2.18 | **FSC vs. SSC Lymphocytes**  95% CI:  1.13-1.92 | **CD56+ cells**  95% CI:  0.89-1.39 | **CD3+CD56+ cells**  95% CI:  1.14-1.94 | **CD3+**  95% CI:  1.14-2.05 | **CD4+**  95% CI:  1.19-2.25 | **CD8+**  95% CI:  1-1.95 | **CD4-CD8-**  95% CI:  0.83-2.47 | **CD20+**  95% CI:  1.25-1.82 |
| --- | --- | --- | --- | --- | --- | --- | --- | --- | --- | --- |
| **PID01** | 0.50 | 0.80 | 1.17 | 1.02 | 1.03 | 1.13 | 1.10 | 0.72 | 1.01 | 1.05 |
| **PID02** | 1.11 | 1.31 | 1.34 | 1.11 | 1.16 | 1.42 | 1.44 | 1.45 | 1.22 | 1.59 |
| **PID03** | 1.41 | 1.74 | 1.27 | 1.15 | 1.21 | 1.30 | 1.33 | 1.27 | 1.13 | 1.51 |
| **PID04** | 3.57 | 4.23 | 2.14 | 2.00 | 2.23 | 1.95 | 1.88 | 2.32 | 1.92 | 4.24 |
| **PID05** | 1.65 | 1.84 | 2.02 | 1.40 | 1.82 | 2.18 | 2.23 | 2.13 | 1.74 | 1.97 |
| **PID06** | 1.48 | 1.64 | 1.54 | 1.36 | 1.57 | 1.51 | 1.51 | 1.47 | 1.45 | 2.55 |
| **PID07** | 1.67 | 2.19 | 1.77 | 1.18 | 1.96 | 1.83 | 1.97 | 1.47 | 2.26 | 1.64 |
| **PID08** | 0.86 | 1.03 | 1.23 | 0.92 | 0.92 | 1.20 | 1.29 | 0.95 | 0.89 | 1.66 |
| **PID09** | 1.61 | 2.19 | 1.33 | 1.14 | 1.37 | 1.33 | 1.34 | 1.32 | 1.24 | 2.00 |
| **PID10** | 2.79 | 2.30 | 2.21 | 1.51 | 2.22 | 2.25 | 2.30 | 2.34 | 1.44 | 1.74 |
| **PID11** | 1.46 | 1.71 | 1.69 | 1.19 | 1.48 | 1.73 | 1.81 | 1.65 | 1.39 | 1.92 |
| **PID12** | 1.39 | 1.56 | 1.50 | 1.00 | 1.50 | 1.62 | 1.72 | 1.53 | 1.64 | 1.58 |
| **PID13** | 1.18 | 1.94 | 1.29 | 1.14 | 1.57 | 1.29 | 1.32 | 1.21 | 0.98 | 1.34 |
| **PID14** | 2.24 | 2.84 | 2.44 | 1.15 | 1.93 | 2.65 | 2.86 | 2.46 | 1.94 | 1.69 |
| **PID15** | 0.44 | 1.14 | 1.44 | 0.99 | 1.52 | 1.49 | 1.64 | 1.26 | 0.98 | 1.54 |
| **PID16** | 3.74 | 2.65 | 1.37 | 1.00 | 0.97 | 1.31 | 1.27 | 1.17 | 5.86 | 1.33 |

**Table S3: CD212/IL-12Rβ1 expression levels (%) and receptor densities (MFI) for each of the cell subsets**.

|  | FSC vs. SSC Monocytes | | CD14+ | | FSC vs. SSC Lymphocytes | | CD56+ | | CD3+CD56+ | | CD3+ | | CD4+ | | CD8+ | | CD4-CD8- | | CD19+ | |
| --- | --- | --- | --- | --- | --- | --- | --- | --- | --- | --- | --- | --- | --- | --- | --- | --- | --- | --- | --- | --- |
|  | %CD212+  95% CI:  22-58 | CD212 MFI  95% CI:  1204-1866 | %CD212+  95% CI:  12-65 | CD212 MFI  95% CI:  1322-2783 | %CD212+  95% CI:  69-90 | CD212 MFI  95% CI:  228-496 | %CD212+  95% CI:  88-96 | CD212 MFI  95% CI:  572-820 | %CD212+  95% CI:  83-93 | CD212 MFI  95% CI:  460-692 | %CD212+  95% CI:  65-90 | CD212 MFI  95% CI:  182-460 | %CD212+  95% CI:  55-87 | CD212 MFI  95% CI:  139-437 | %CD212+  95% CI:  71-92 | CD212 MFI  95% CI:  240-545 | %CD212+  95% CI:  81-92 | CD212 MFI  95% CI:  303-501 | %CD212+  95% CI:  68-88 | CD212 MFI  95% CI:  158-416 |
| PID01 | 29.80 | 1257 | 35.00 | 1331 | 78.60 | 272 | 82.90 | 423 | 77.90 | 346 | 80.10 | 258 | 74.90 | 234 | 80.80 | 284 | 75.00 | 247 | 80.90 | 269 |
| PID02 | 32.70 | 1365 | 63.20 | 2088 | 85.20 | 357 | 91.80 | 532 | 88.10 | 457 | 82.80 | 264 | 78.30 | 238 | 83.90 | 295 | 81.00 | 291 | 81.30 | 259 |
| PID03 | 9.91 | 921 | 23.20 | 1198 | 81.40 | 278 | 84.10 | 448 | 83.00 | 373 | 82.20 | 256 | 79.40 | 251 | 81.70 | 268 | 78.30 | 252 | 83.90 | 264 |
| PID04 | 39.40 | 1402 | 71.80 | 2382 | 70.60 | 191 | 77.80 | 409 | 69.50 | 274 | 73.30 | 185 | 68.90 | 174 | 76.10 | 226 | 78.20 | 249 | 68.60 | 165 |
| PID05 | 19.70 | 969 | 39.80 | 1445 | 70.60 | 198 | 72.50 | 395 | 66.00 | 245 | 72.10 | 180 | 68.30 | 171 | 70.90 | 195 | 66.80 | 171 | 71.50 | 182 |
| PID06 | 51.60 | 1745 | 66.10 | 2355 | 75.00 | 245 | 84.10 | 471 | 78.00 | 362 | 76.30 | 227 | 71.20 | 202 | 80.20 | 284 | 72.90 | 228 | 71.30 | 195 |
| PID07 | 31.60 | 1270 | 56.80 | 1952 | 78.50 | 261 | 86.60 | 558 | 82.40 | 411 | 81.10 | 261 | 77.50 | 242 | 84.50 | 321 | 80.40 | 289 | 71.80 | 192 |
| PID08 | 55.90 | 1949 | 63.90 | 2230 | 76.70 | 257 | 80.00 | 430 | 83.30 | 423 | 78.80 | 244 | 70.20 | 191 | 90.10 | 363 | 85.60 | 291 | 72.00 | 186 |
| PID09 | 15.80 | 1016 | 13.70 | 1069 | 75.60 | 231 | 83.10 | 470 | 80.00 | 401 | 78.80 | 235 | 75.20 | 221 | 79.70 | 265 | 77.00 | 285 | 68.10 | 170 |
| PID10 | 60.90 | 1939 | 86.20 | 3000 | 76.00 | 228 | 88.80 | 616 | 81.80 | 532 | 77.20 | 205 | 71.90 | 175 | 80.40 | 257 | 81.00 | 302 | 68.30 | 136 |
| PID11 | 20.30 | 1096 | 25.40 | 1207 | 77.30 | 247 | 86.80 | 544 | 82.70 | 417 | 77.80 | 217 | 71.90 | 194 | 80.50 | 259 | 79.50 | 262 | 80.10 | 242 |
| PID12 | 21.90 | 991 | 26.80 | 1088 | 71.00 | 202 | 81.70 | 459 | 69.20 | 282 | 72.80 | 184 | 69.40 | 178 | 69.90 | 190 | 67.00 | 190 | 64.40 | 153 |
| PID13 | 9.82 | 754 | 10.60 | 943 | 68.40 | 184 | 75.70 | 445 | 72.00 | 296 | 70.80 | 175 | 66.30 | 161 | 70.50 | 203 | 66.80 | 184 | 65.80 | 154 |
| PID14 | 30.80 | 1320 | 39.50 | 1519 | 52.80 | 112 | 75.20 | 479 | 84.40 | 521 | 73.30 | 255 | 69.50 | 244 | 76.70 | 301 | 67.00 | 217 | 62.50 | 165 |
| PID15 | 45.00 | 1616 | 68.50 | 2200 | 85.00 | 395 | 91.10 | 625 | 89.40 | 581 | 84.40 | 352 | 81.10 | 329 | 92.10 | 516 | 81.90 | 320 | 85.60 | 316 |
| PID16 | 42.40 | 1527 | 51.30 | 1801 | 69.00 | 171 | 78.00 | 438 | 76.00 | 407 | 70.30 | 155 | 65.00 | 137 | 71.80 | 190 | 71.40 | 195 | 66.60 | 134 |

**Table S4: Fold change in pSTAT4 for each of the cell subsets**.

| **Patient ID** | **FSC vs. SSC Monocytes**  95% CI:  1.44-2.1 | **CD14+**  95% CI:  1.27-2.15 | **FSC vs. SSC Lymphocytes**  95% CI:  1.03-1.6 | **NK cells**  95% CI:  1.92-2.96 | **NKT cells**  95% CI:  1.14-1.75 | **CD3+**  95% CI:  1.03-1.73 | **CD4+**  95% CI:  1.04-1.47 | **CD8+**  95% CI:  1.16-2.25 | **CD4-CD8-**  95% CI:  0.98-3.05 | **CD20+**  95% CI:  0.95-1.63 |
| --- | --- | --- | --- | --- | --- | --- | --- | --- | --- | --- |
| **PID01** | 1.58 | 1.58 | 1.45 | 1.83 | 1.55 | 1.39 | 1.32 | 1.48 | 1.74 | 2.15 |
| **PID02** | 1.47 | 1.20 | 1.48 | 1.32 | 1.48 | 1.57 | 1.44 | 1.93 | 1.20 | 1.35 |
| **PID03** | 1.51 | 1.25 | 1.10 | 1.07 | 0.95 | 1.10 | 1.07 | 1.25 | 1.07 | 1.15 |
| **PID04** | 3.92 | 2.41 | 3.49 | 9.21 | 2.34 | 3.06 | 2.68 | 4.95 | 3.62 | 14.14 |
| **PID05** | 1.26 | 1.01 | 1.07 | 1.19 | 0.98 | 1.13 | 1.04 | 1.25 | 1.53 | 0.82 |
| **PID06** | 1.84 | 1.75 | 1.38 | 2.33 | 1.33 | 1.36 | 1.27 | 1.50 | 1.38 | 1.31 |
| **PID07** | 2.48 | 2.35 | 1.79 | 1.50 | 1.28 | 1.71 | 1.53 | 1.74 | 5.02 | 1.42 |
| **PID08** | 1.32 | 1.35 | 1.33 | 1.51 | 1.12 | 1.32 | 1.33 | 1.24 | 1.81 | 1.17 |
| **PID09** | 1.57 | 1.40 | 1.32 | 1.43 | 1.15 | 1.38 | 1.30 | 1.67 | 1.22 | 1.18 |
| **PID10** | 0.91 | 0.93 | 1.66 | 2.17 | 1.62 | 1.58 | 1.68 | 1.85 | 1.58 | 1.52 |
| **PID11** | 1.06 | 1.02 | 0.88 | 2.26 | 0.95 | 0.83 | 0.84 | 0.82 | 0.85 | 1.00 |
| **PID12** | 2.05 | 2.13 | 1.60 | 1.53 | 1.50 | 1.61 | 1.56 | 1.76 | 1.75 | 1.43 |
| **PID13** | 1.53 | 1.68 | 0.75 | 1.88 | 0.82 | 0.69 | 0.68 | 0.69 | 0.83 | 1.10 |
| **PID14** | 0.94 | 0.97 | 1.63 | 1.56 | 1.24 | 1.54 | 1.32 | 1.79 | 3.63 | 1.36 |
| **PID15** | 1.13 | 1.04 | 0.96 | 1.18 | 1.05 | 0.94 | 0.92 | 1.07 | 0.88 | 0.98 |
| **PID16** | 1.11 | 1.23 | 1.44 | 1.43 | 0.51 | 1.28 | 1.09 | 1.64 | 0.57 | 1.20 |

**Table S5: Cytokine-induced cytokine production values for all participants.**

| **Patient ID** | **IFN-γ production assay** | | | | **IL-12 production assay** | | | |
| --- | --- | --- | --- | --- | --- | --- | --- | --- |
|  | **NIL (pg/mL)**  95% CI: 0-0 | **PHA (pg/mL)**  (NIL values subtracted)  95% CI:  45-686 | **PHA+IL-12 (pg/mL)**  (NIL values subtracted)  95% CI:  433-735 | **Net IL-12-induced IFN-γ**  **production (pg/mL)**  95% CI: 49-444 | **NIL (pg/mL)**  95% CI: 0-0 | **PHA (pg/mL)**  (NIL values subtracted)  95% CI:  0.04-2.66 | **PHA+IFN-γ (pg/mL)**  (NIL values subtracted)  95% CI: 8.9-21 | **Net IFN-γ-induced**  **IL-12 production (pg/mL)**  95% CI: 8.94-18 |
| **PID01** | 0.00 | 70.00 | 425.40 | 355.40 | 0.00 | 0.26 | 0.69 | 0.43 |
| **PID02** | 0.00 | 57.49 | 1512.29 | 1454.80 | 0.00 | 0.90 | 22.14 | 21.24 |
| **PID03** | 0.00 | 480.56 | 620.00 | 139.43 | 0.00 | 18.63 | 38.83 | 20.20 |
| **PID04** | 0.00 | 15.85 | 562.76 | 546.91 | 0.00 | 1.84 | 36.90 | 35.06 |
| **PID05** | 0.00 | 68.75 | 755.31 | 686.56 | 0.00 | 0.19 | 2.47 | 2.28 |
| **PID06** | 11.54 | 25.67 | 74.23 | 48.56 | 0.00 | 0.05 | 1.12 | 1.07 |
| **PID07** | 0.00 | 5.49 | 299.91 | 294.42 | 0.00 | 0.21 | 4.81 | 4.61 |
| **PID08** | 10.31 | -6.68 | 25.55 | 32.23 | 0.00 | 0.27 | 3.72 | 3.44 |
| **PID09** | 0.00 | 19.57 | 399.54 | 379.97 | 0.00 | 0.11 | 1.64 | 1.54 |
| **PID10** | 0.00 | 566.17 | 795.34 | 229.16 | 0.00 | 0.82 | 10.94 | 10.12 |
| **PID11** | 0.00 | 2.09 | 106.01 | 103.93 | 0.00 | 0.87 | 30.49 | 29.62 |
| **PID12** | 0.00 | 279.06 | 764.92 | 485.86 | 0.00 | 1.86 | 25.48 | 23.61 |
| **PID13** | 0.00 | 12.32 | 215.79 | 203.47 | 0.00 | 0.04 | 2.79 | 2.75 |
| **PID14** | 0.00 | 27.92 | 901.82 | 873.90 | 0.00 | 0.46 | 17.38 | 16.92 |
| **PID15** | 0.00 | 23.09 | 425.11 | 402.02 | 0.00 | 0.41 | 29.18 | 28.77 |
| **PID16** | 0.00 | 24.03 | 312.83 | 288.80 | 0.00 | 0.24 | 8.98 | 8.74 |
